# Atlas of non-pathological solitary or asymmetrical skeletal muscle uptake in [^18^F]FDG-PET

**DOI:** 10.1007/s11604-022-01264-3

**Published:** 2022-03-28

**Authors:** Tomohiko Yamane, Yohji Matsusaka, Kenji Fukushima, Akira Seto, Ichiro Matsunari, Ichiei Kuji

**Affiliations:** 1 Department of Molecular Imaging Research, Kobe City Medical Center General Hospital, 2-1-1 Minatojima-Minamimachi, Chuo-ku, Kobe, 650-0047 Japan; 2 Department of Nuclear Medicine, Saitama Medical University International Medical Center, Hidaka, Japan; 3 Department of Radiology and Nuclear Medicine, Fukushima Medical University, Fukushima, Japan; 4 Division of Nuclear Medicine, Department of Radiology, Saitama Medical University Hospital, Moroyama, Japan

**Keywords:** 2-Deoxy-2-[^18^F]fluoro-d-glucose ([^18^F]FDG), Skeletal muscle, Physiological uptake, Positron emission tomography/computed tomography (PET/CT)

## Abstract

Positron emission tomography (PET) using 2-deoxy-2-[^18^F]fluoro-d-glucose ([^18^F]FDG) is widely used in oncology and other fields. In [^18^F]FDG PET images, increased muscle uptake is observed owing exercise load or muscle tension, in addition to malignant tumors and inflammation. Moreover, we occasionally observe non-pathological solitary or unilateral skeletal muscle uptake, which is difficult to explain the strict reason. In most cases, we can interpret them as not having pathological significance. However, it is important to recognize such muscle uptake patterns to avoid misdiagnoses with pathological ones. Therefore, the teaching point of this pictorial essay is to comprehend the patterns of solitary or asymmetrical skeletal muscle uptake seen in routine [^18^F]FDG-PET scans. As an educational goal, you will be able to mention muscles where intense physiological [^18^F]FDG uptake can be observed, differentiate between physiological muscle uptake and lesion, and discuss with any physicians or specialists about uncertain muscle uptake.

## Introduction

As a glucose analog tracer in positron emission tomography (PET), 2-deoxy-2-[^18^F]fluoro-d-glucose ([^18^F]FDG) has been widely used in patients with malignant tumors as well as inflammatory diseases, cardiology, and neurology. It is well known that muscle [^18^F]FDG uptake increases after exercise or in a state of high serum insulin levels, including failure to fast [1–3]. In addition, we occasionally observed intense [^18^F]FDG uptake in a single or a group of muscles in routine [^18^F]FDG PET examinations, even though the patient did not have any history of recent exercise. We can easily interpret them as not of pathological significance, although it is usually difficult to explain the detailed mechanism of such mysterious uptake in most cases. They may be caused by muscle hypertonus, involuntary movement, or specific habits of the patient. In clinical practice, we certainly do not need to determine the exact reasons, because we can easily understand that it is a kind of physiological and meaningless uptake. Nevertheless, it is important to recognize such increased uptake patterns in muscles to avoid misdiagnosis of actual pathological lesions. For instance, intense muscle uptake that appears after chemotherapy has a fear of misdiagnosis as a recurrent lesion, especially by physicians who are not familiar with [^18^F]FDG PET. In this pictorial essay, such intense muscle uptake patterns observed on [^18^F] FDG-PET are shown as maximum intensity projection or fused PET/CT images.

## Head and neck

**Fig. 1:**
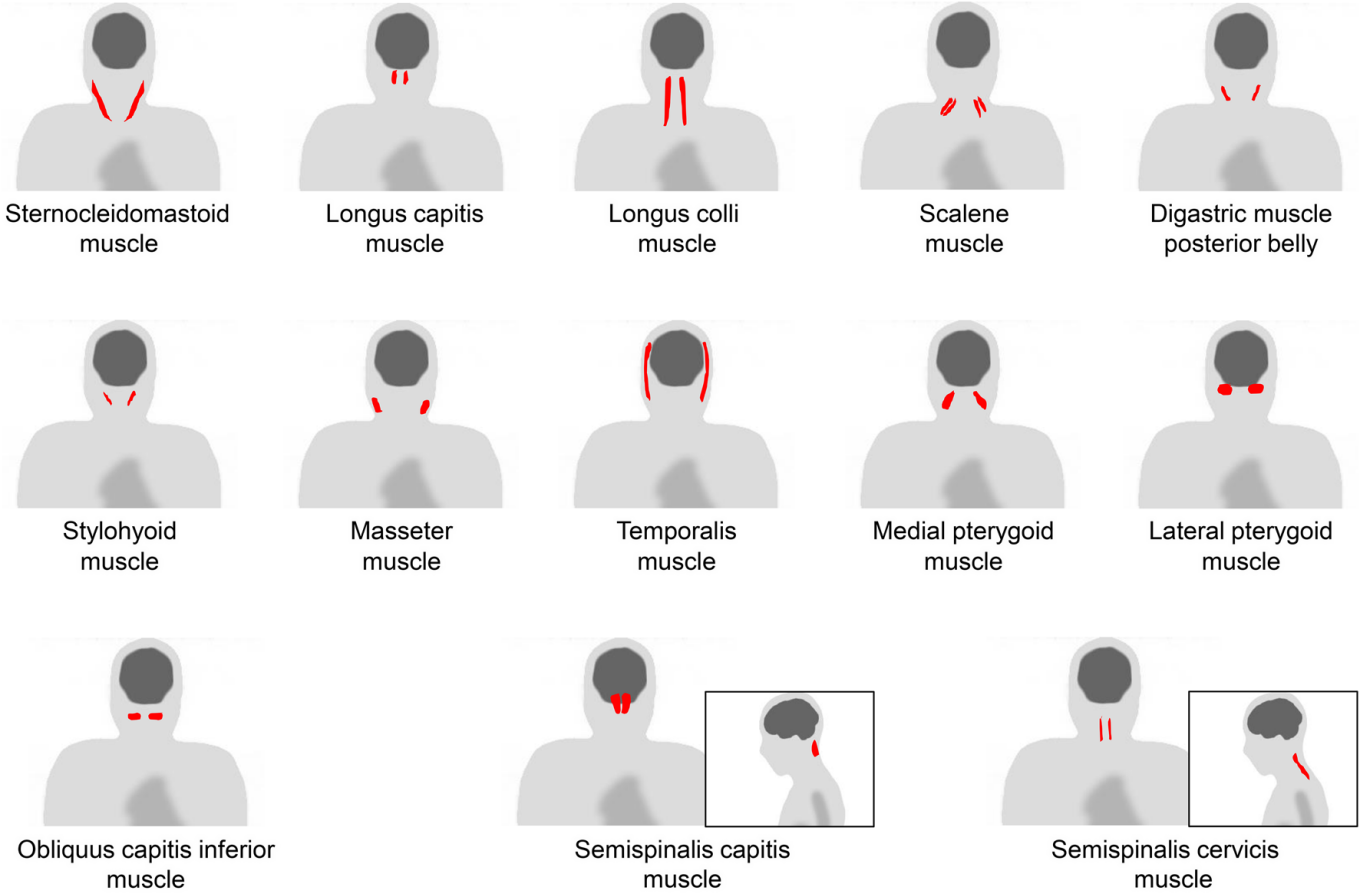
Muscles’ schemas in the head and neck area

**Fig. 2:**
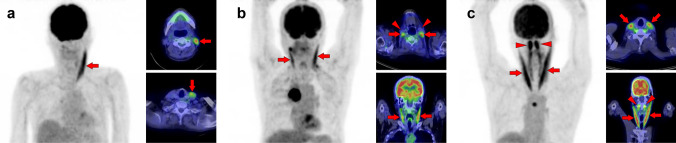
*Sternocleidomastoid muscle.* The sternocleidomastoid is one of the most frequently visualized muscles on [^18^F]FDG PET. However, unilateral isolated ones may be occasionally hard to interpret for beginners (**a**, arrow). There are two branches in the sternocleidomastoid muscles, which originate from the sternum and the clavicle. The sternal branch is more commonly visualized but is rare for the clavicular branch (**b**, arrows) without uptake in the sternal branch (**b**, arrowheads). The longus capitis and longus colli muscles (**c**, arrowheads) are often observed together with the sternocleidomastoid muscle (**c**, arrows)

**Fig. 3:**
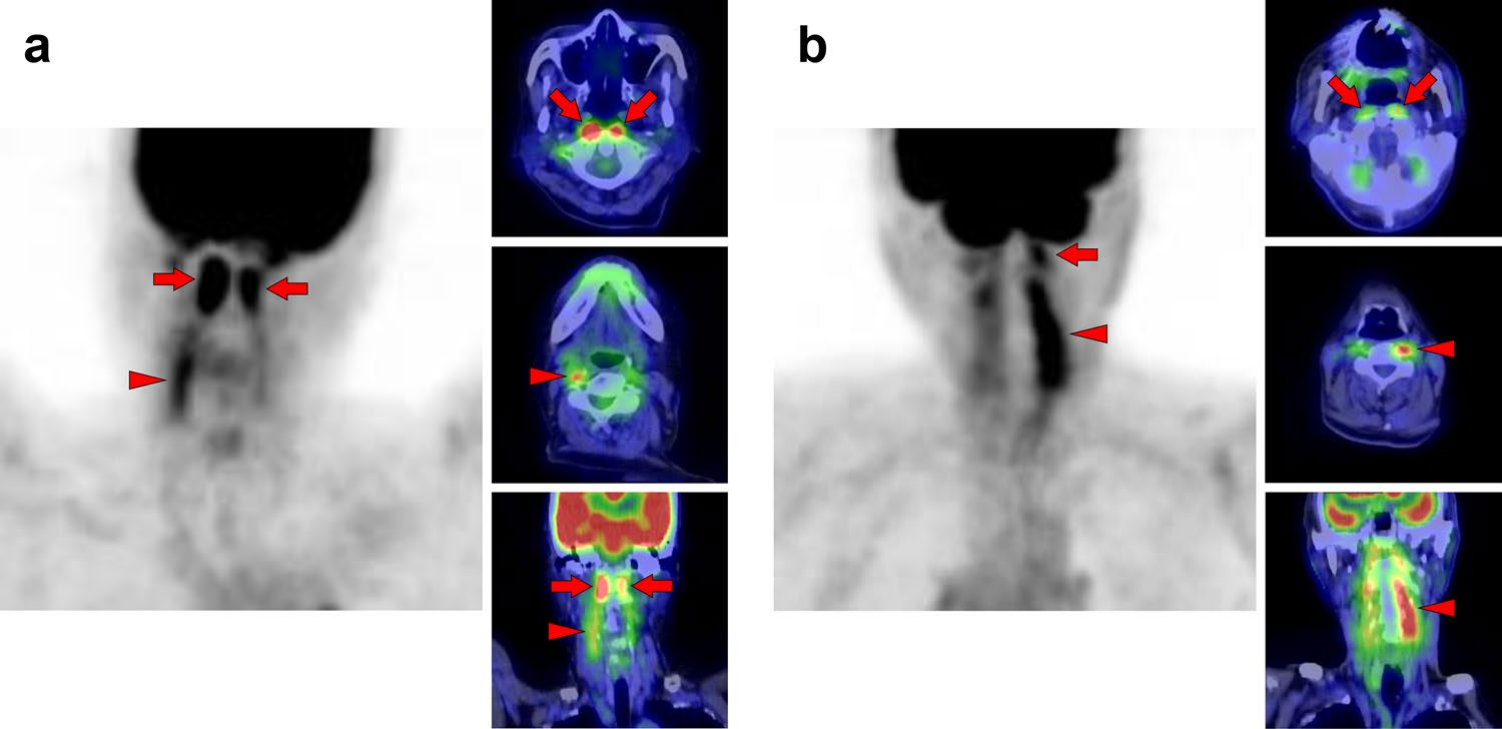
*Longus capitis muscle and longus colli muscle.* Although longus capitis muscle (arrows) and longus colli muscle (arrowheads) tend to be visualized simultaneously, there are both cases of longus capitis dominant pattern (**a**) and longus colli dominant pattern (**b**), and the uneven lateral distribution can be observed

**Fig. 4:**
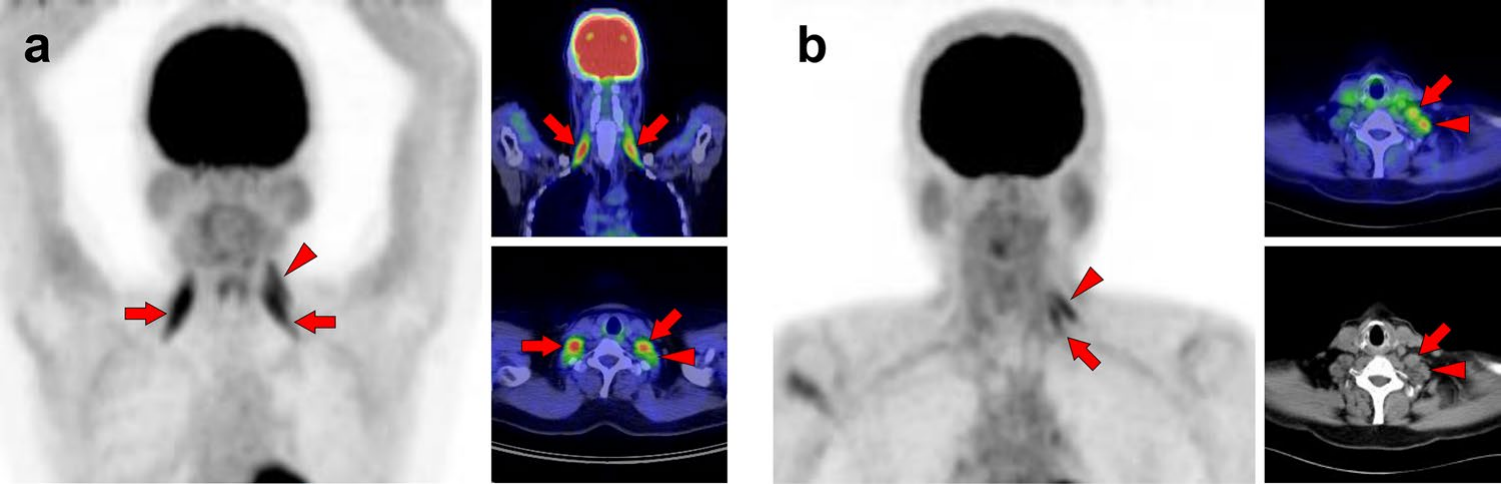
*Scalene muscle.* Both bilateral (**a**) and unilateral (**b**) uptake in scalene muscle is frequently observed when weak uptake is included. While the scalene muscle is divided into anterior, middle, and posterior parts, the frequency of visualization in the anterior scalene muscle (arrows) is high and followed by middle and posterior scalene muscles (arrowheads). We need to differentiate them with brown fat and [^18^F]FDG-avid lymph node, and the key is whether it is along the direction of the muscle. The scalene muscle is included in the group of the respiratory muscles, and the visualization of the other respiratory muscles or the history of respiratory impairment may be helpful for the evaluation

**Fig. 5:**
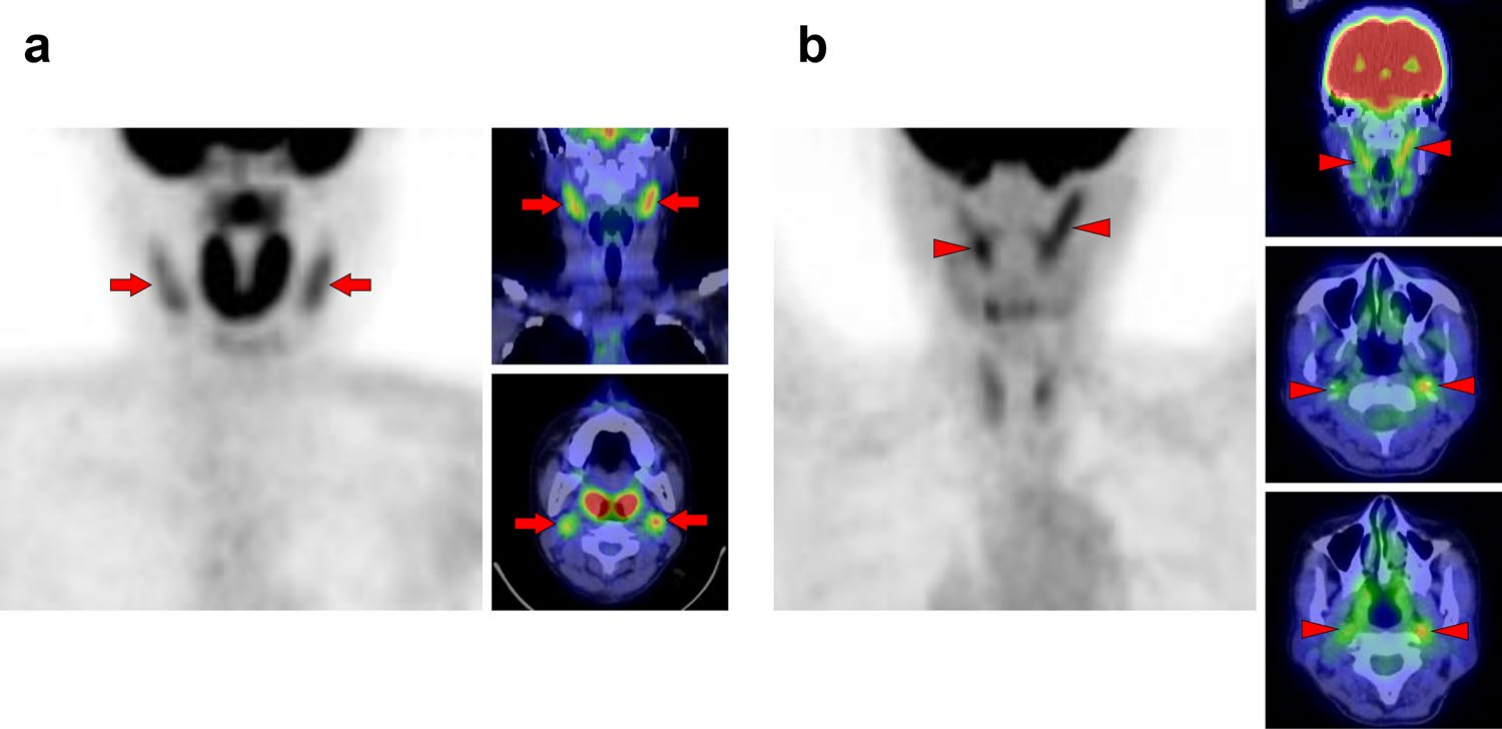
*Suprahyoid muscles.* The suprahyoid muscles are located on the head side of the hyoid bone. Increased [^18^F]FDG uptake is often observed in the posterior belly of the digastric muscle (a, arrows) and the stylohyoid muscle (b, arrowheads); both belong to the suprahyoid muscle. These muscles are occasionally visualized as nodular in shape and may be misdiagnosed as a lymph-node uptake at the upper internal jugular area

**Fig. 6:**
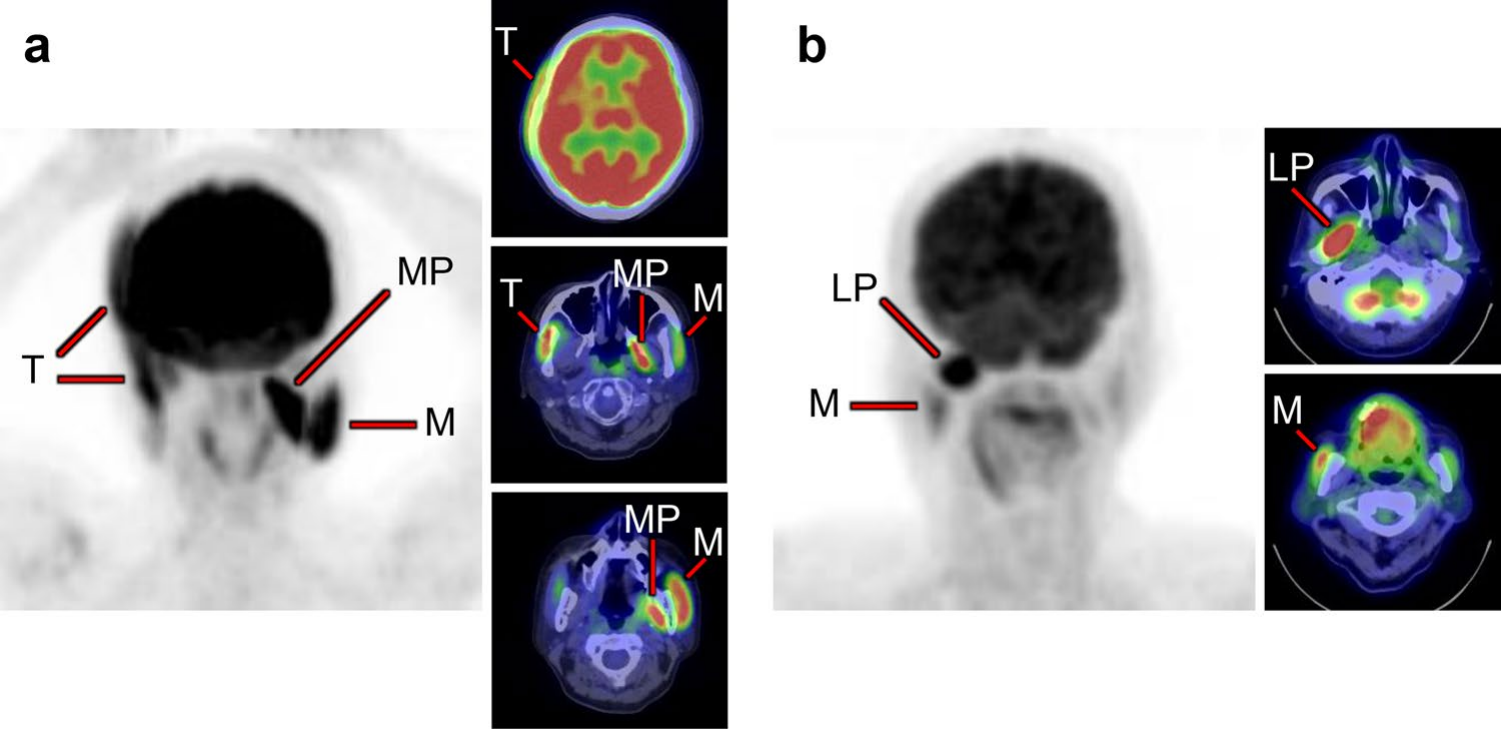
*Muscles of mastication.* Increased [^18^F]FDG uptake in masticatory muscles, consisting of the masseter (M), temporalis (T), medial pterygoid (MP), and lateral pterygoid (LP), is well known to be observed when chewing gum, but there are also various causes such as involuntary movements and bruxism. A few masticatory muscles are often depicted at the same time, and rarely are they seen alone. They are usually visualized as asymmetrical. A case with increased [^18^F]FDG uptake at the right temporalis, left masseter, and left medial pterygoid (**a**), and a case with uptake at the right lateral pterygoid and right masseter (**b**) are shown

**Fig. 7:**
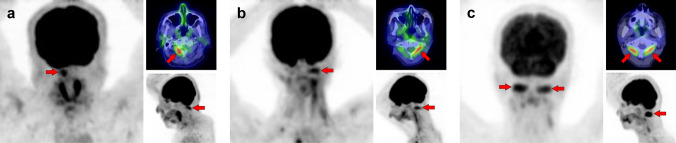
*Obliquus capitis inferior muscle.* We can frequently find faint [^18^F]FDG uptake when closely observed, but occasionally experience surprisingly strong uptake in the obliquus capitis inferior muscle. It may be misdiagnosed as a metastatic lesion if it is visualized unilaterally. Therefore, it would be important to understand the pattern of this muscle. Three cases with unilateral uptake (**a**, **b**) and bilateral uptake (**c**) of the obliquus capitis inferior muscle (arrows) are shown

**Fig. 8:**
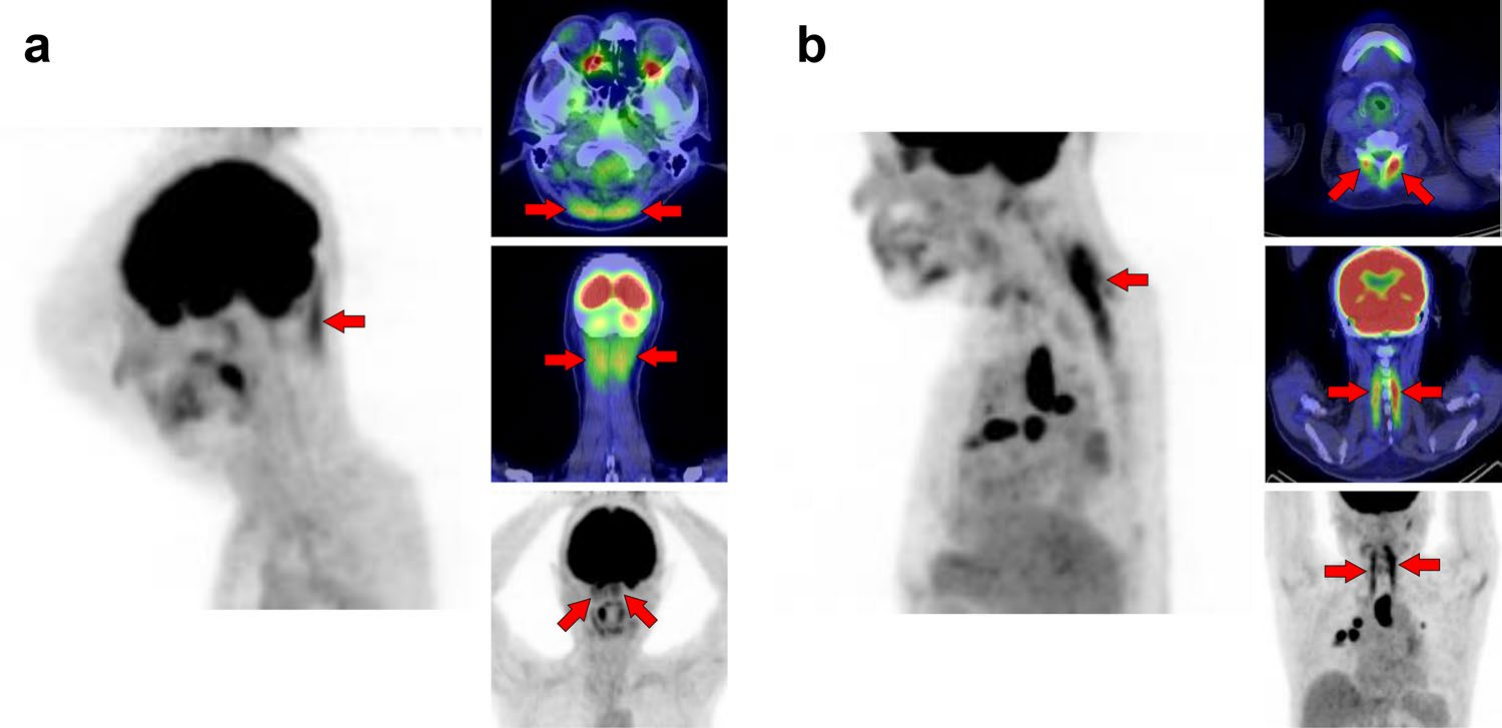
*Semispinalis capitis muscle and semispinalis cervicis muscle.* Although strong [^18^F]FDG uptake is occasionally observed, there is usually no specific problem in differentiating from pathological accumulation. Cases of increased uptake in semispinalis capitis muscle (**a**, arrows) and semispinalis cervicis muscle (**b**, arrows) are shown

When dealing with the head and neck region in [^18^F]FDG-PET interpretation, the physiological uptake of the organs in this area should be noted [4, 5]. In addition, vascular inflammation and dental metal artifacts visualized on PET/CT images may interrupt precise evaluation [6, 7]. It should also be noted that non-pathological muscle uptake is frequently visualized after surgery for head and neck cancer [8]. The extraocular muscles are usually visualized as intense uptake. Moreover, patients who chew gum should also be considered [1]. Figure 1 illustrates a schema of the muscles in the head and neck region where physiological accumulation is frequently observed. We introduced the sternocleidomastoid muscle (Fig. 2), longus capitis and longus colli muscles (Fig. 3), scalene muscle (Fig. 4), suprahyoid muscles (Fig. 5), mastication muscles (Fig. 6), obliquus capitis inferior muscle (Fig. 7), and semispinalis capitis muscle and semispinalis cervicis muscle (Fig. 8).

## Chest and upper arm

**Fig. 9:**
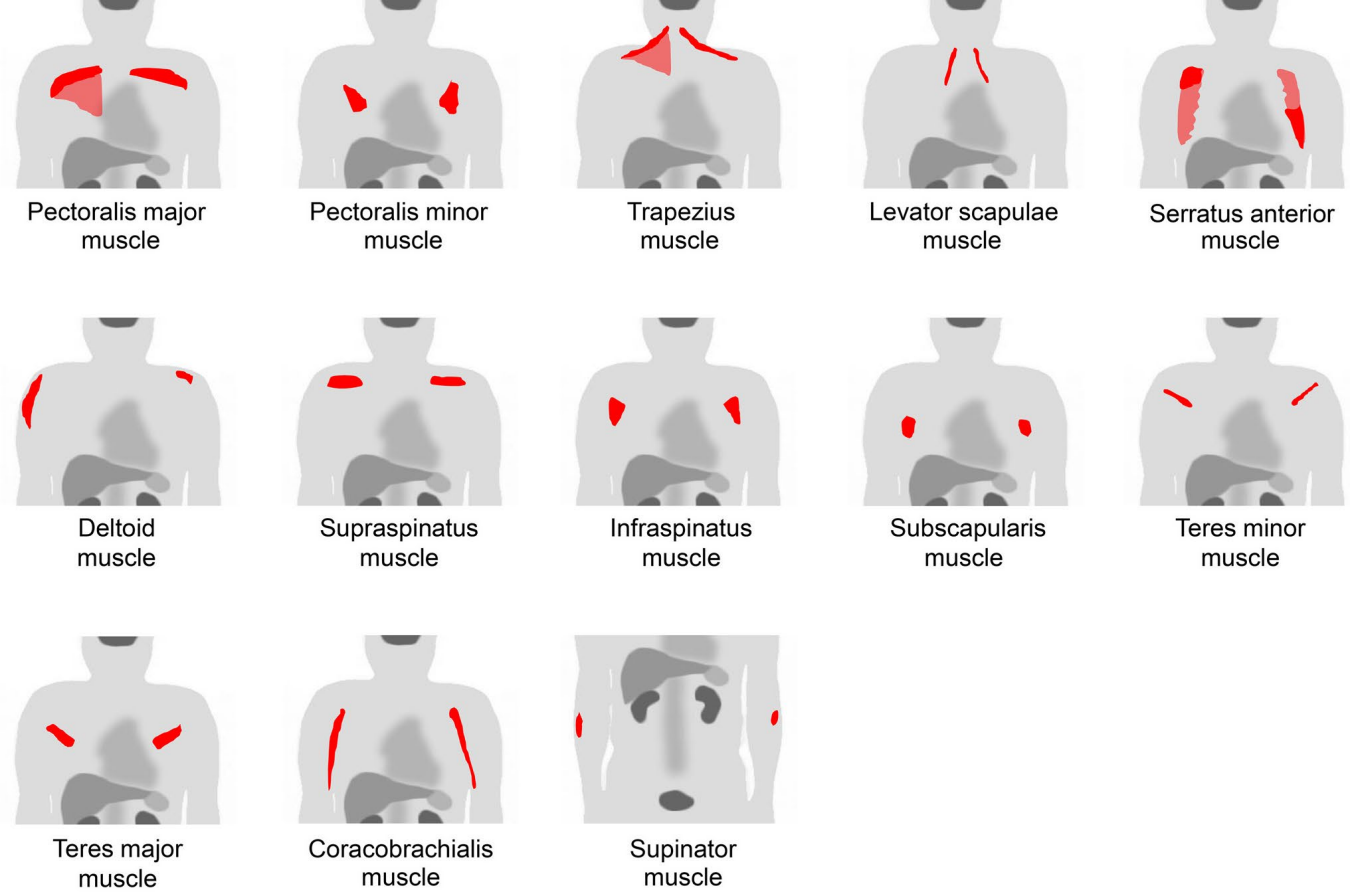
Muscle schemas in the chest and upper arm area

**Fig. 10:**
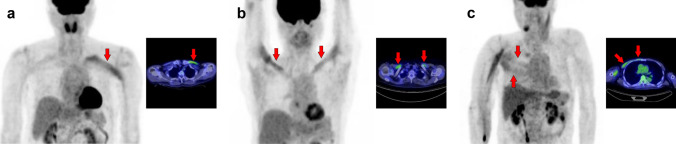
*Pectoralis major muscle.* The pectoralis major generally consists of a clavicular part (**a**, **b**, arrows) and a sternal part (**c**, arrows); [^18^F]FDG uptake can be observed in both or separately. When the clavicular part is depicted alone, it may be misinterpreted as vascular inflammation

**Fig. 11:**
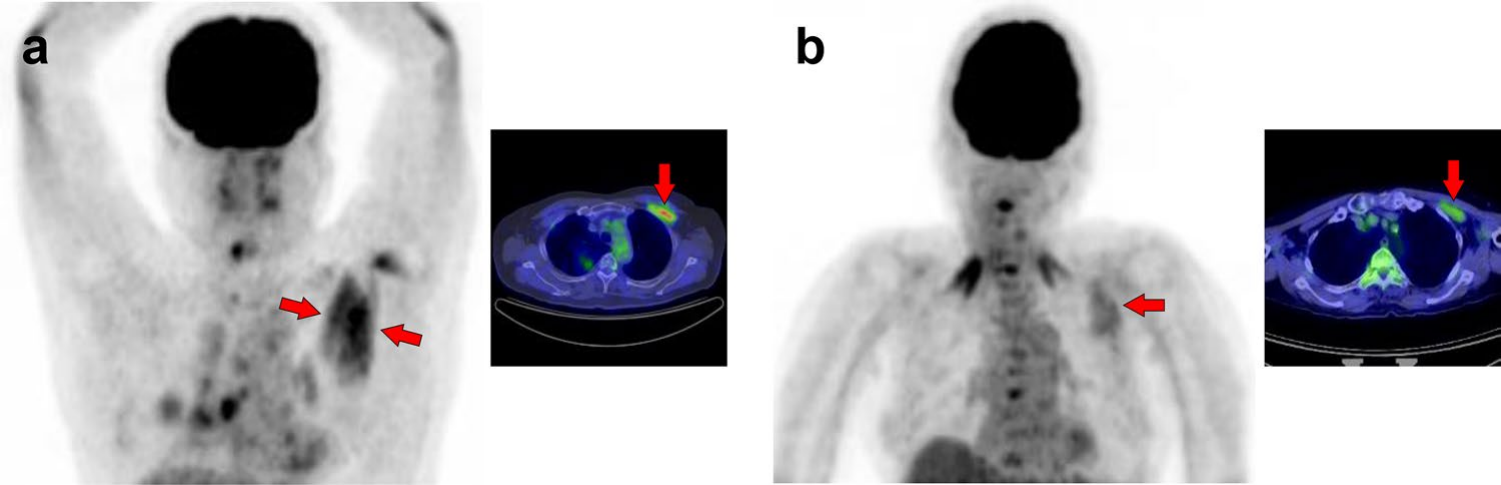
*Pectoralis minor muscle.* The pectoralis minor can be observed without visualization of the pectoralis major (**a**, **b**, arrows). Conversely, uptake in the other muscles is occasionally accompanied, and we can find the uptake in the supraspinatus muscle in case (**a**) and in scalene muscle in case (**b**). CT should also be evaluated carefully when a cardiac pacemaker is implanted, as it can be confused with postoperative inflammation or attenuation correction errors due to CT metal artifacts

**Fig. 12:**
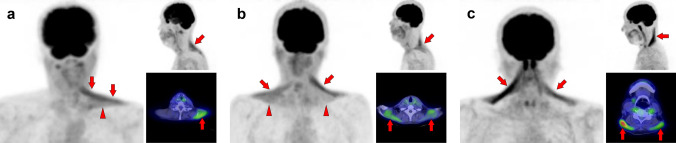
*Trapezius muscle.* The trapezius (**a**–**c**, arrows) is divided into upper, middle, and lower parts. The typical uptake pattern of the trapezius seems to be observed by a strong uptake in the middle part with a weak uptake in the lower part. Visualization of the upper fibers seems rare, but it can be depicted with other neck muscles such as the longus capitis and longus colli (**c**)

**Fig. 13:**
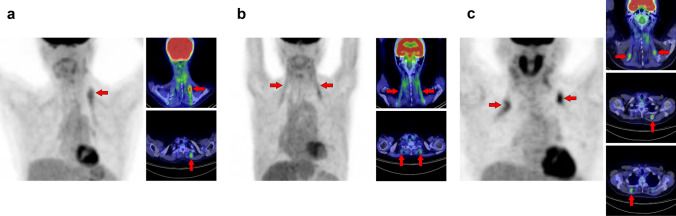
*Levator scapulae muscle.* The levator scapulae (**a**–**c**, arrows) can present with a long cephalocaudal uptake pattern. Occasionally, a nodular uptake pattern is visualized (**c**), which may be mistaken for lymph-node lesions or activation of brown fat

**Fig. 14:**
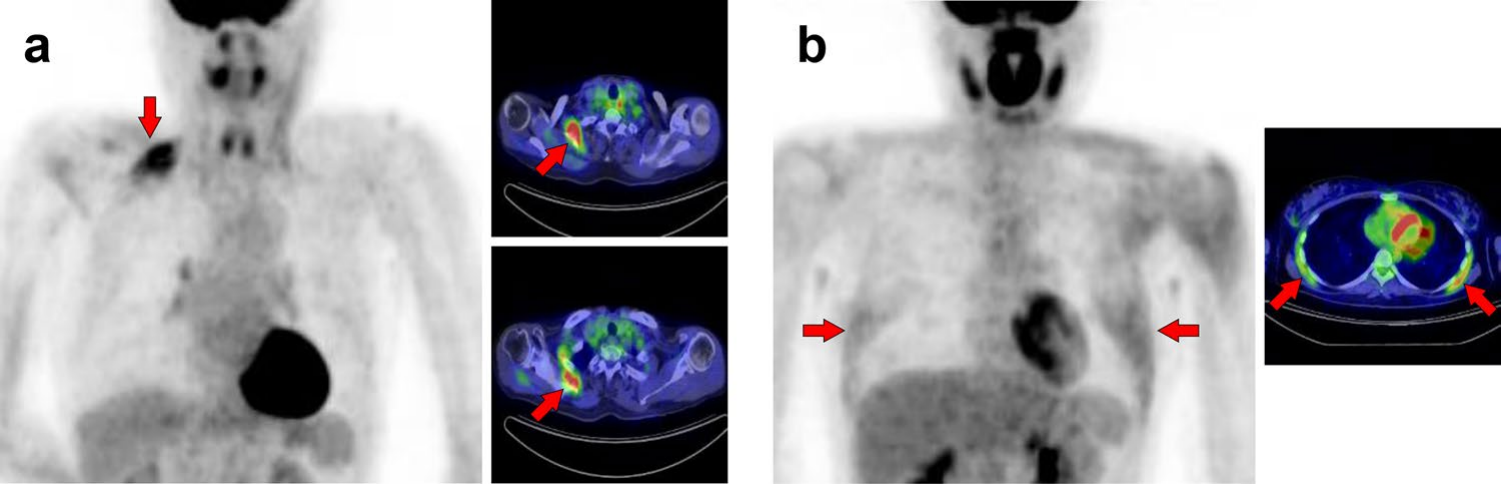
*Serratus anterior muscle.* The serratus anterior muscle (**a**, **b**, arrows) can be divided into upper, middle, and lower parts. Increased uptake in the middle and lower parts observed after exercise or in hyperglycemia (**b**) is easy to interpret, whereas high uptake in the upper fibers alone (**a**) may require differentiation from malignant lesions. It is known that [^18^F]FDG accumulates in elastofibroma dorsi, which should be distinguished from muscle uptake in the serratus anterior and subscapularis muscles

**Fig. 15:**
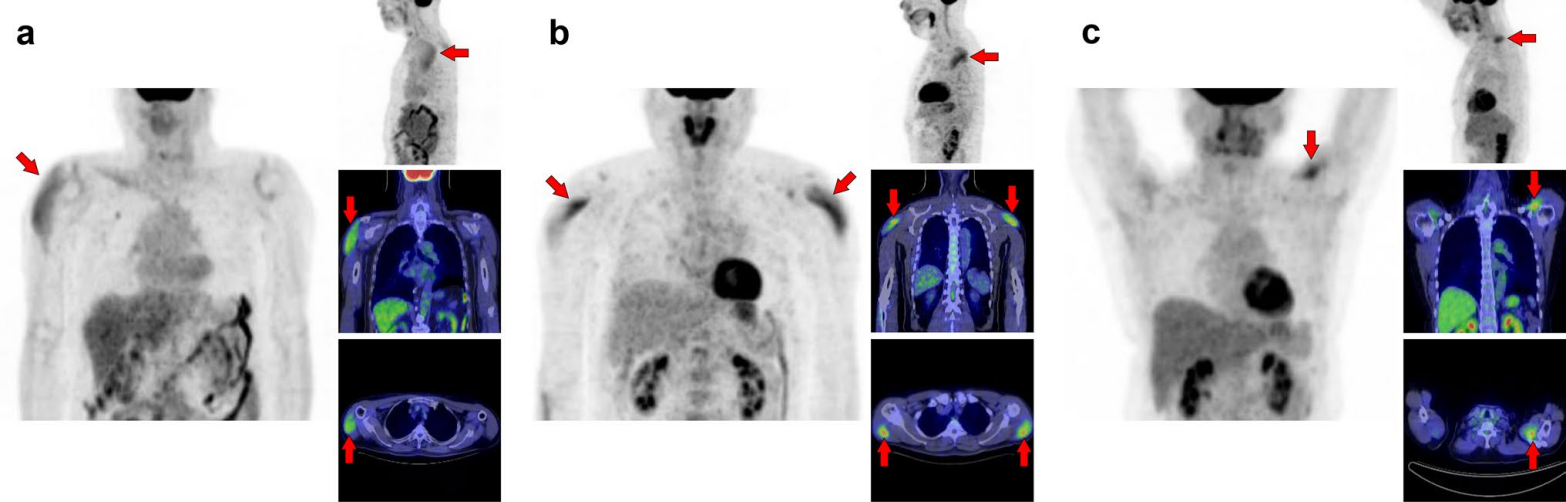
*Deltoid muscle.* The deltoid (**a**–**c**, arrows) has three origins: the anterior originating at the outer 1/3 of the clavicle (**c**), the middle originating at the acromion (**a**), and the posterior originating at the spine of scapula (**b**). Increased uptake in the deltoid can be observed as limited in one of these areas

**Fig. 16:**
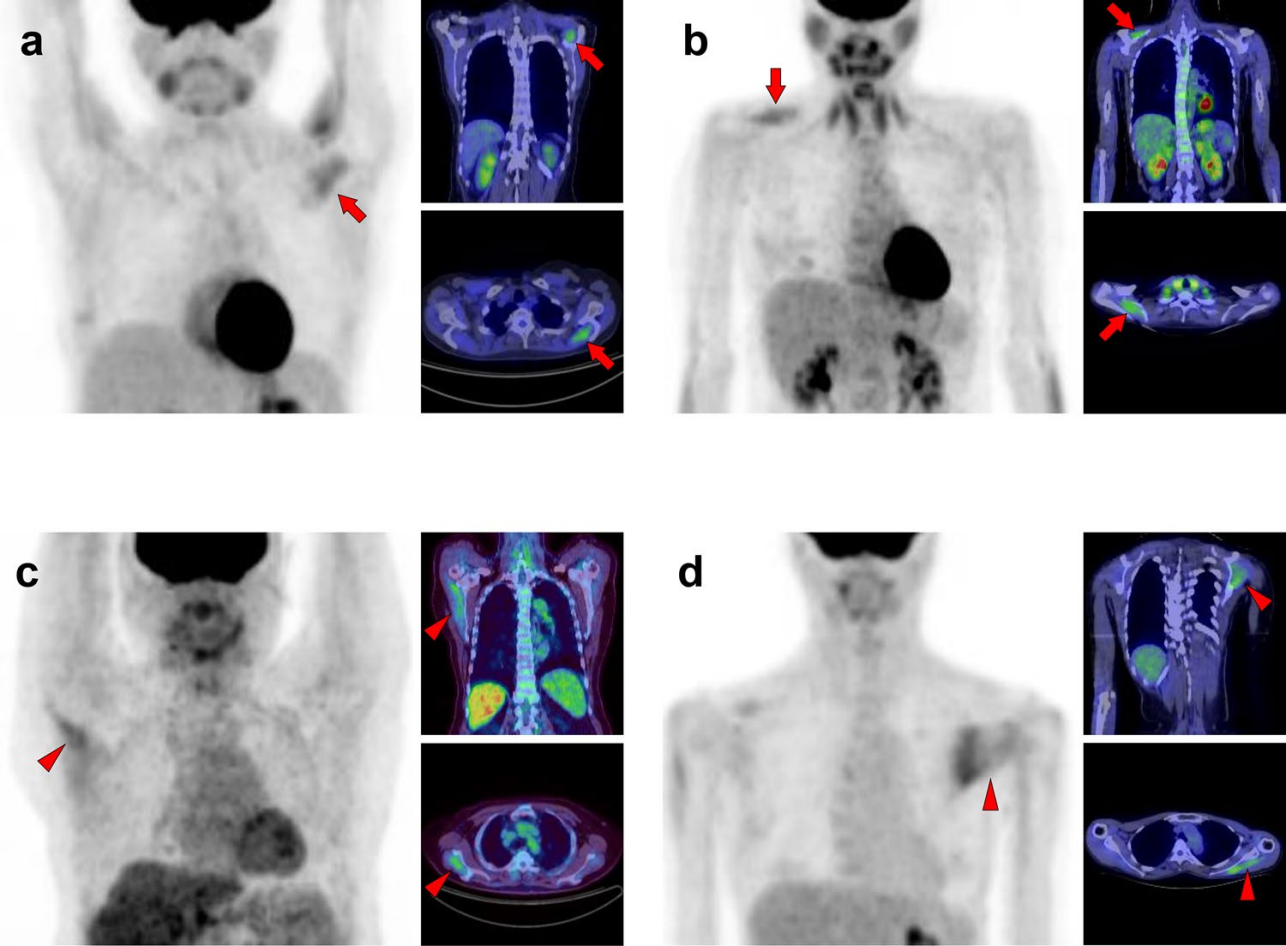
*Supraspinatus muscle and infraspinatus muscle.* The supraspinatus muscle (**a**, **b**, arrows) and the infraspinatus muscles (**c**, **d**, arrowheads) are frequently visualized as solitary or unilateral

**Fig. 17:**
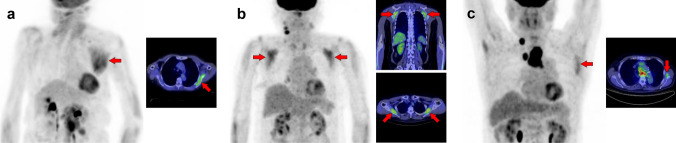
*Subscapularis muscle.* We can observe a triangle-shaped distribution of subscapularis muscle (**a**, **b**, arrows) when [^18^F]FDG accumulates into the whole of the muscle. However, there are some cases where [^18^F]FDG uptake is found only in the lower parts, and it appears to be on the lateral side when the upper extremity is elevated (**c**, arrows)

**Fig. 18:**
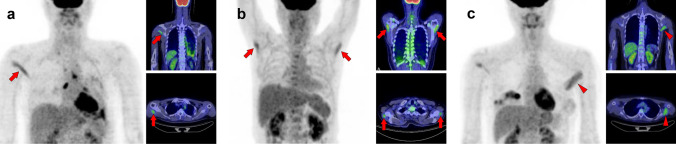
*Teres minor muscle and teres major muscle.* The teres minor is one of the most frequently depicted muscles, and we can find both unilateral and bilateral cases (**a**, **b**, arrows). While it cannot be a problem in differentiating it from malignant lesions, we should carefully identify the uptake region to distinguish it from inflammation of the shoulder joint. The uptake of teres major can be observed alone, but this is rare compared to the teres minor (**c**, arrowheads)

**Fig. 19:**
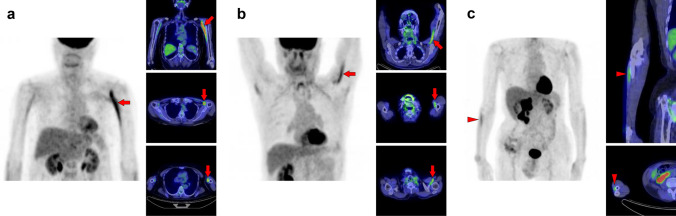
*Coracobrachialis muscle and supinator muscle.* The coracobrachialis muscle can be visualized occasionally (**a**, **b**, arrows). However, untrained readers may misinterpret this linear image along the arm as retained [^18^F]FDG in vein. The supinator is also frequently visualized in [^18^F]FDG PET images (**c**, arrowheads). We can often observe other muscles in the arm in various forms, while it is not easy to simplify

In the thoracic region, respiratory muscles should be considered [1, 3, 9]. The respiratory muscles include the diaphragm and intercostal muscles. During labored breathing, the sternocleidomastoid and scalene are also used for inspiration, and the abdominal muscles are used for expiration. In addition, the site of [^18^F]FDG injection [10, 11] or recent vaccination history [12] should be considered when evaluating the upper extremities. Vascular inflammation and metal artifacts from cardiac implantable devices also interrupt evaluation in the chest area [6, 7]. We should attempt to obtain information on the habit of muscle training or posture during the waiting time after [^18^F]FDG injection. In addition, muscle uptake should be carefully distinguished from benign lesions that mimic muscles, such as elastofibroma dorsi [13]. Figure 9 illustrates a schema of muscles in the chest and upper arm regions where physiological accumulation is frequently observed. We illustrated the pectoralis major muscle (Fig. 10), pectoralis minor muscle (Fig. 11), trapezius muscle (Fig. 12), levator scapulae muscle (Fig. 13), serratus anterior muscle (Fig. 14), deltoid muscle (Fig. 15), supraspinatus and infraspinatus muscles (Fig. 16), subscapularis muscle (Fig. 17), teres minor and teres major muscles (Fig. 18), and coracobrachialis and supinator muscles (Fig. 19).

## Abdomen, pelvis, and femur

**Fig. 20:**
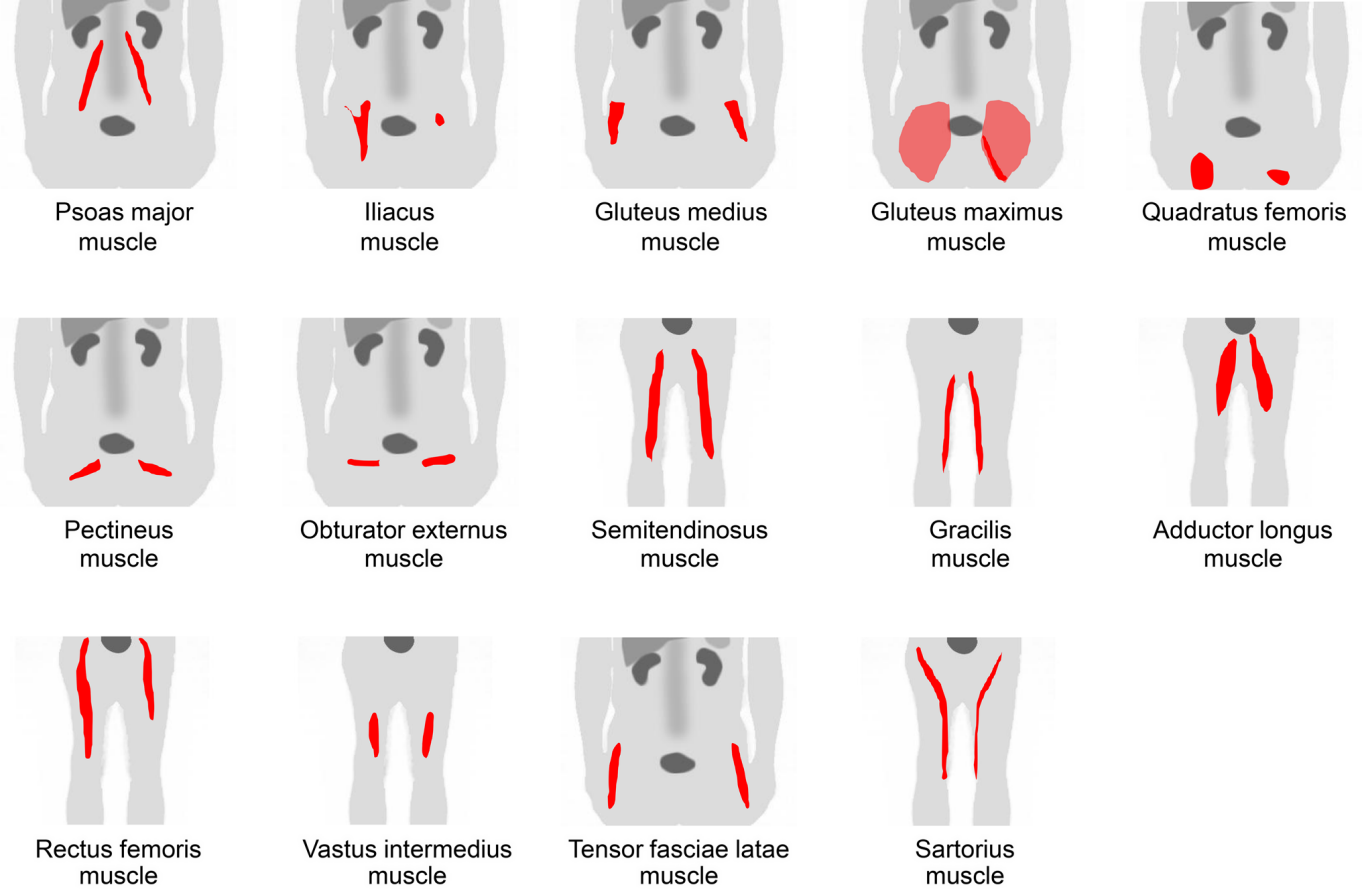
Muscle schemas in the abdomen, pelvis, and femur area

**Fig. 21:**
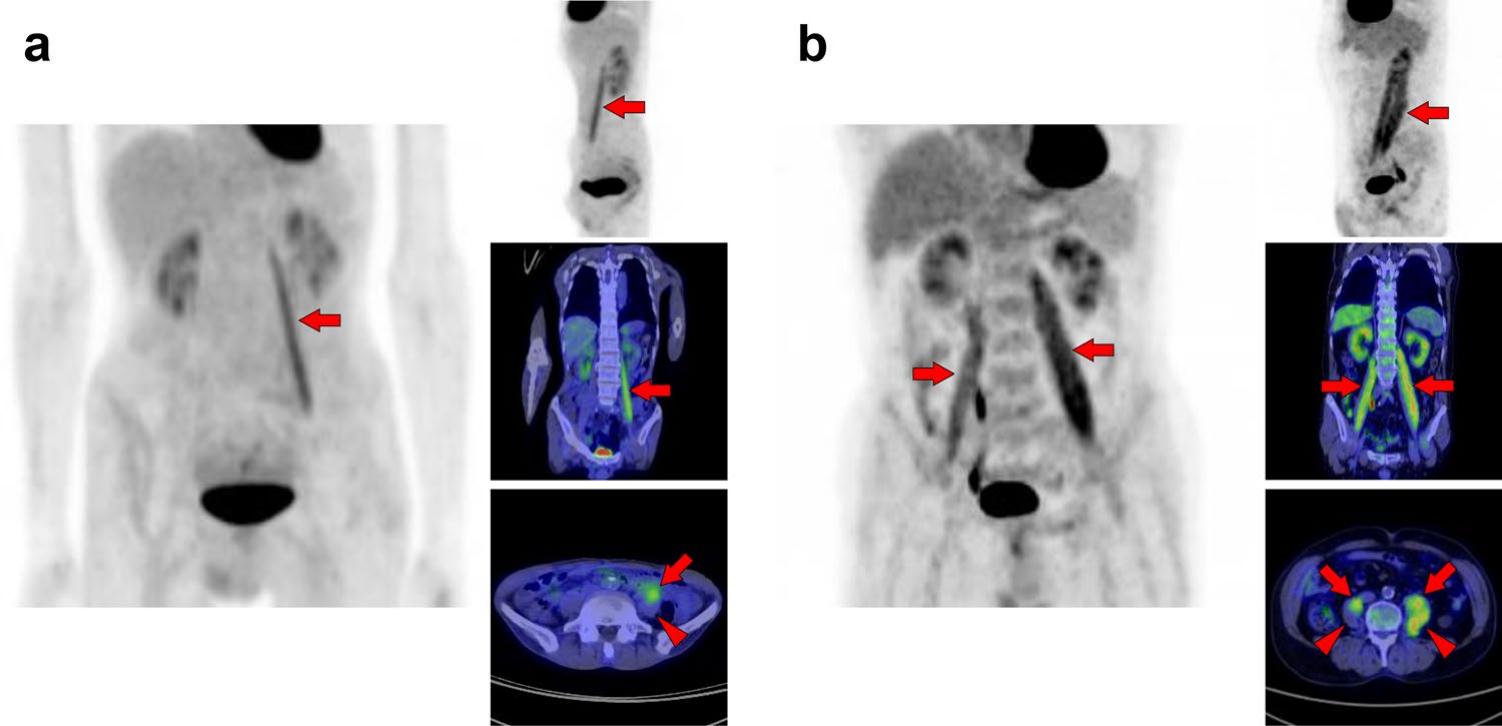
*Psoas major muscle.* [^18^F]FDG uptake in the psoas major is often visualized bilaterally but can also be unilateral or have strong laterality (**a**, **b**). The psoas major has superficial (arrows) and deep (arrowheads) parts, and uptake in the superficial tends to be dominant

**Fig. 22:**
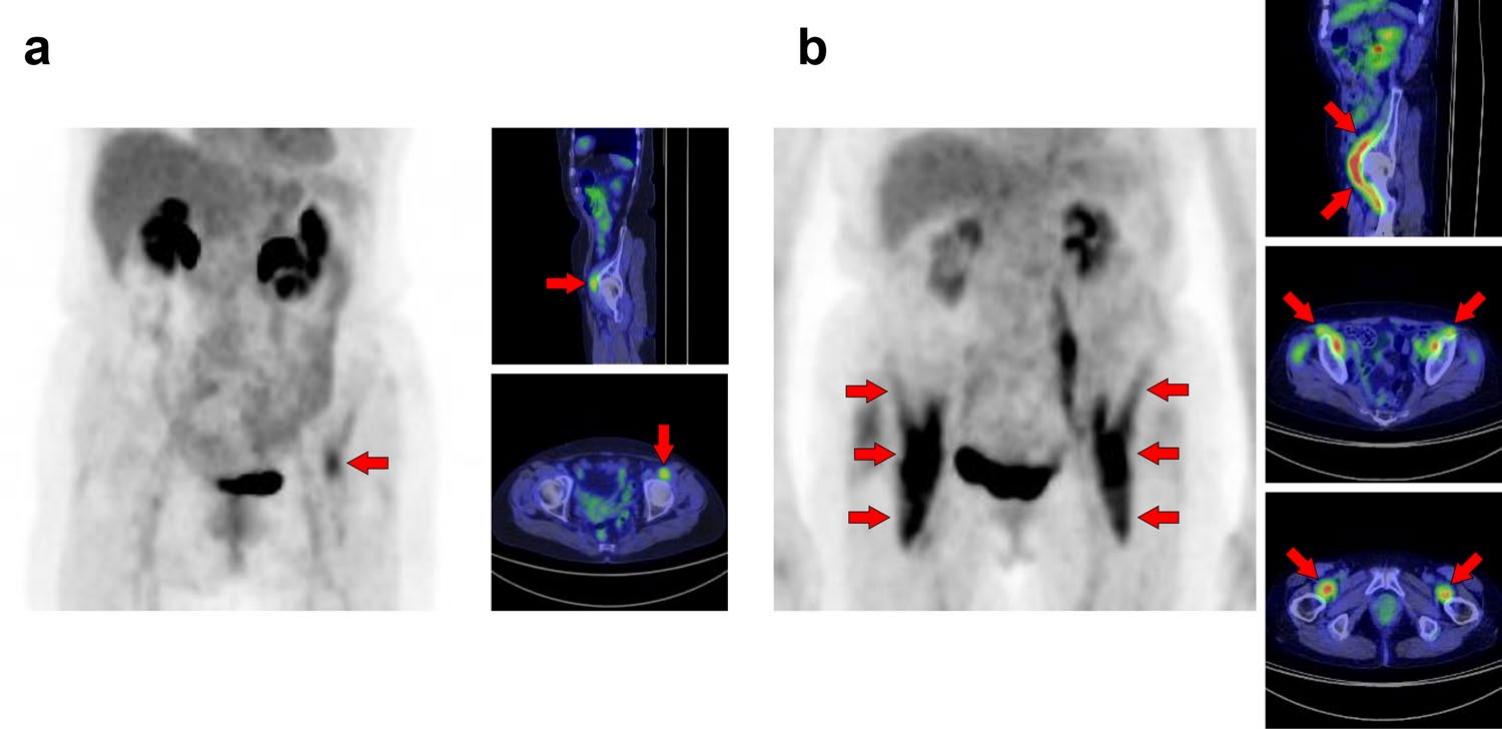
*Iliacus muscle.* Nodular uptake in the iliopsoas muscle can be found around the ventral side of the femoral head (**a**, arrows). In addition, the entire iliopsoas muscle is occasionally observed strongly under stress (**b**, arrows)

**Fig. 23:**
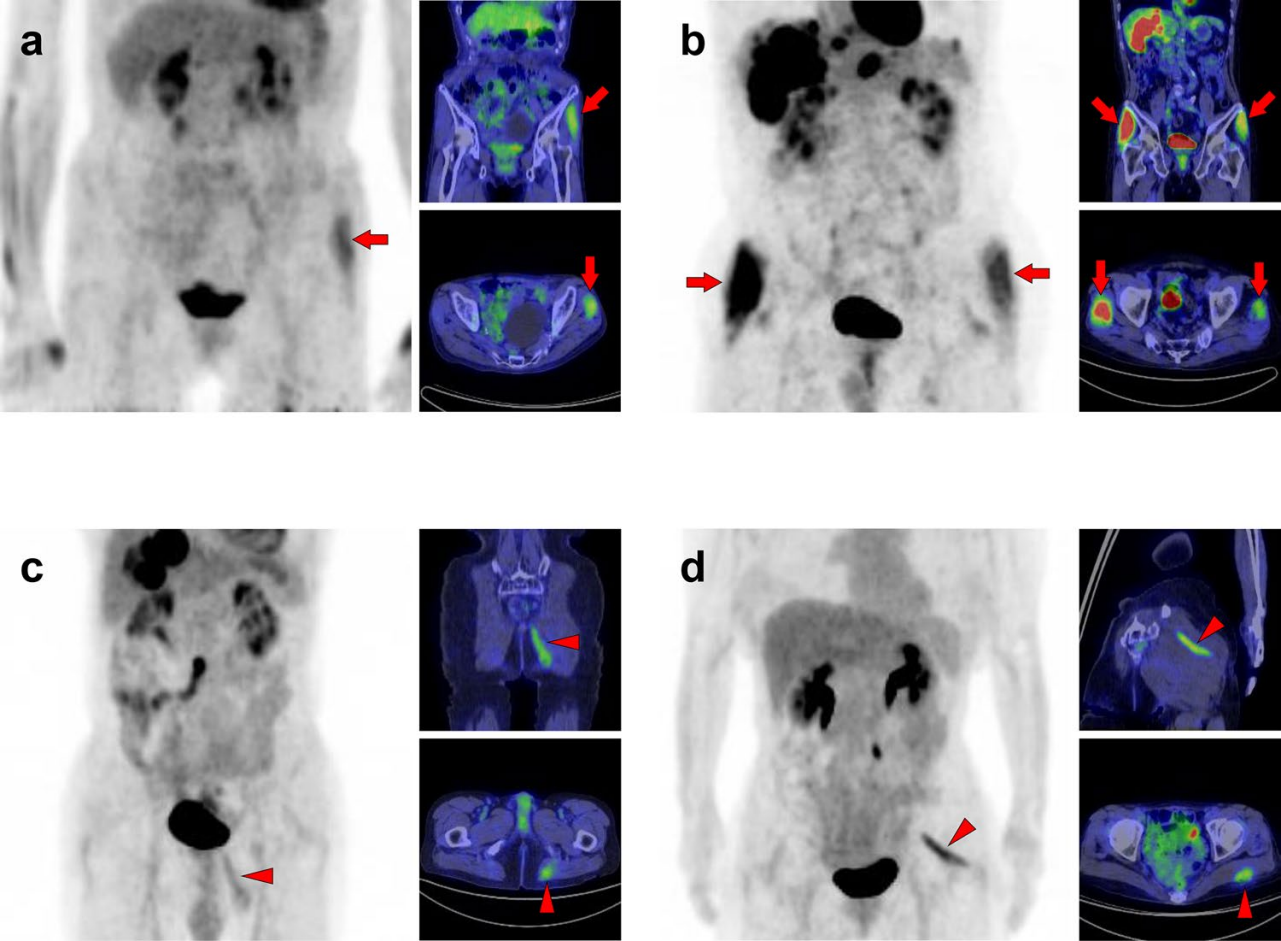
*Gluteus medius muscle and gluteus maximus muscle.* The gluteus medius (**a**, **b**, arrows), where non-pathologically increased [^18^F]FDG uptake is frequently observed among the gluteus, is composed of anterior, middle, and posterior parts. The anterior parts located in the lateral side are most often found to be with uptake. In contrast, the gluteus maximus muscle is often visualized entirely with other skeletal muscles after exercise or in a high-insulin state. However, localized uptake may be observed limited in the lower parts located medially (**c**, arrowheads). Since the gluteus maximus muscle is often chosen for intramuscular injection, we should consider the possibility of a medically induced uptake (**d**, arrowheads)

**Fig. 24:**
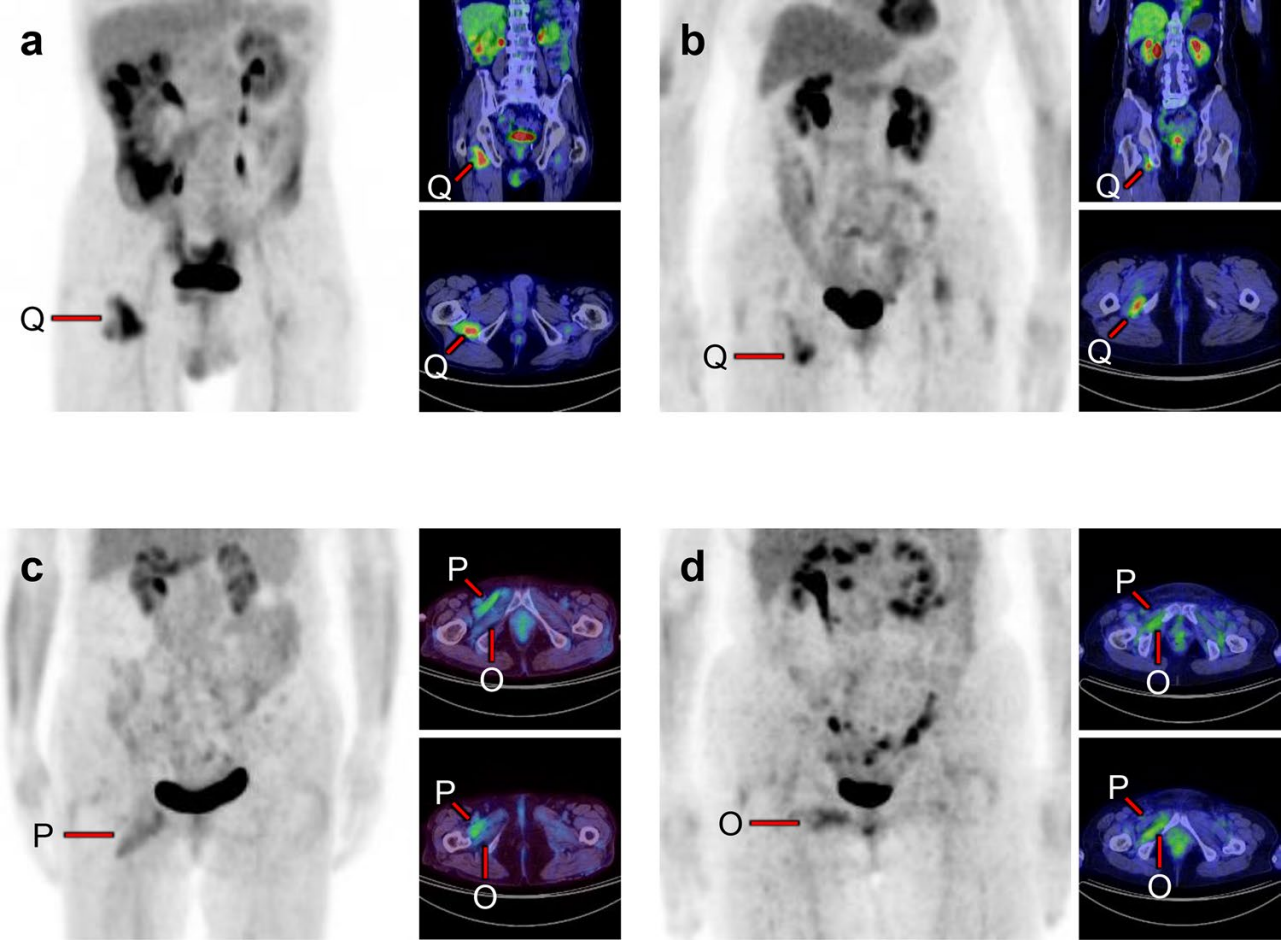
*Quadratus femoris muscle, pectineus muscle, and obturator externus muscle.* We occasionally find a solitary increased uptake in the quadratus femoris (**a**, **b**; Q), while the inflammatory uptake caused by osteoarthritis in the hip and surrounding tissues may interrupt clear visualization. The pectineus muscle (**c**, P) originates from the pectineal line of the pubis and inserts into the pectineal line and the linea aspera of the femur. The external obturator muscle (**d**, O) originates from the obturator foramen and the obturator membrane and inserts into the trochanteric fossa of the femur. Although not so frequent, isolated uptakes may be seen in these muscles

**Fig. 25:**
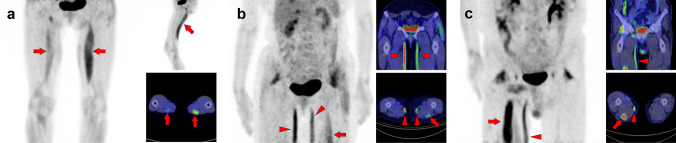
S*emitendinosus muscle and gracilis muscle.* The biceps femoris, semitendinosus, and semimembranosus muscles are the major flexor muscle groups known as hamstrings. Among them, [^18^F]FDG accumulates more frequently in the semimembranosus (**a**–**c**, arrows). Moreover, the gracilis muscle (**b**, **c**, arrowheads) is occasionally seen as a specific strong uptake, which may look like a ureteral catheter at first glance. The gracilis muscle tends to be visualized with other muscles, such as the semimembranosus muscle

**Fig. 26:**
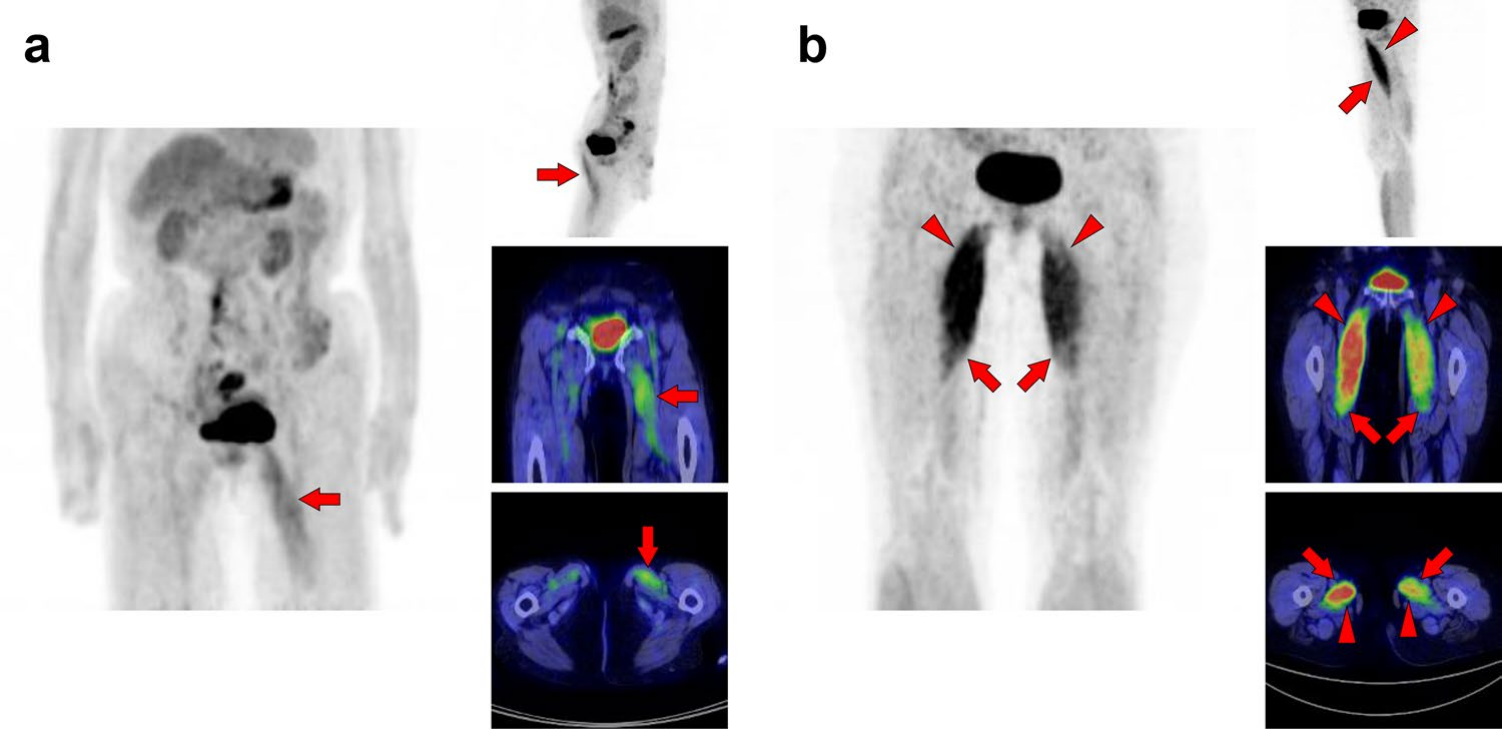
*Adductor longus muscle and adductor brevis muscle.* Uptake in the adductor femoris muscles can be visualized, and we occasionally observe that in the adductor longus (arrows) and adductor brevis (arrowheads). We present a case in which only an adductor longus muscle is displayed (**a**) and a case in which the adductor longus and brevis muscles are simultaneously displayed (**b**)

**Fig. 27:**
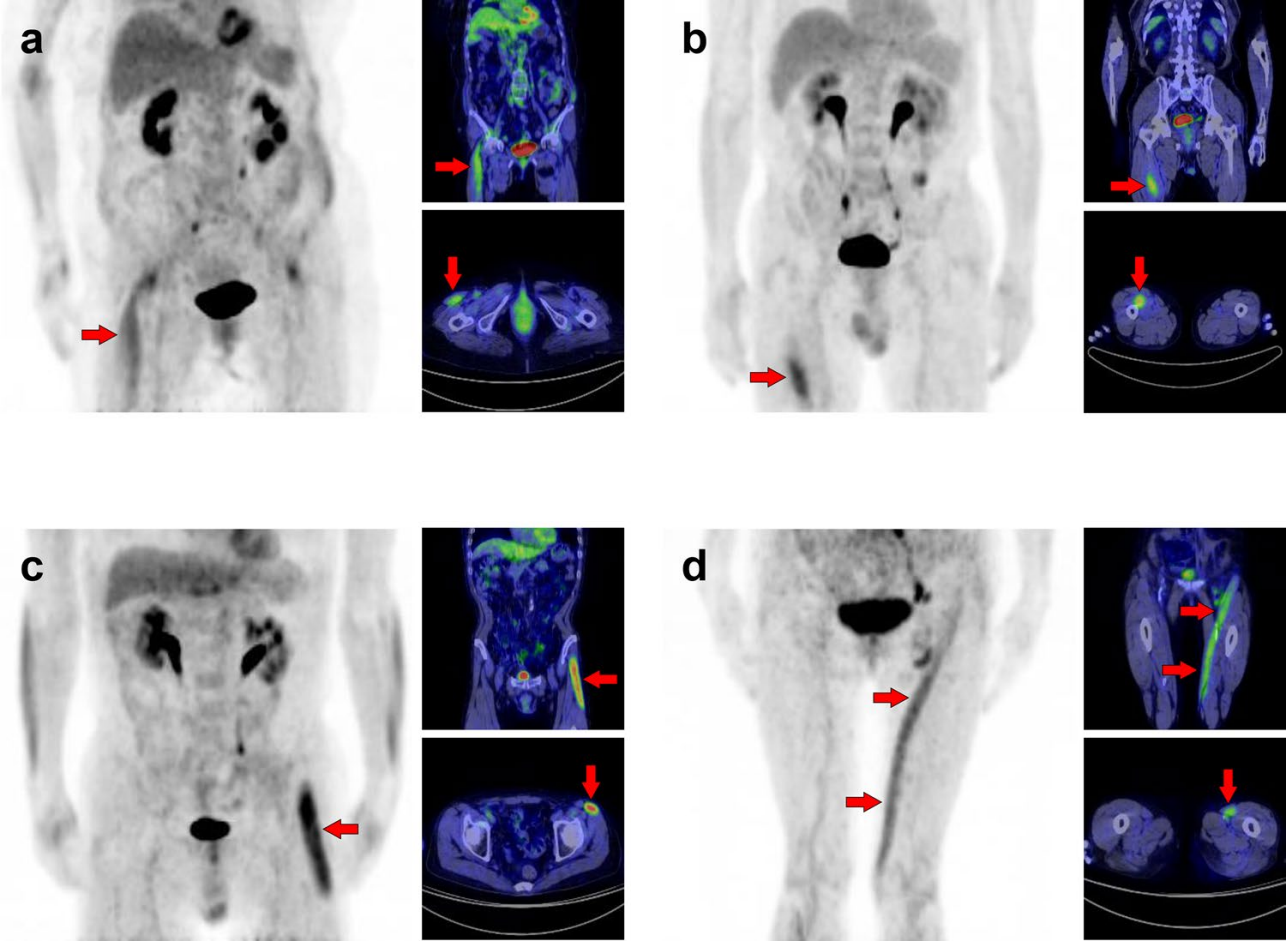
*Quadriceps femoris muscles, tensor fasciae latae muscle, and sartorius muscle.* The quadriceps consists of four muscles: rectus femoris, vastus medialis, vastus lateralis, and vastus intermedius. Unilateral uptake in a single muscle can be observed, and here is a case in which the rectus femoris muscle is visualized (**a**, arrows) and the vastus medialis muscle is visualized (**b**, arrows). Rare but intense uptake can be also observed in the tensor fasciae latae muscle (**c**, arrows). Moreover, uptake in the sartorius muscle is visualized as a long line running obliquely down the thigh (**d**, arrows)

**Fig. 28:**
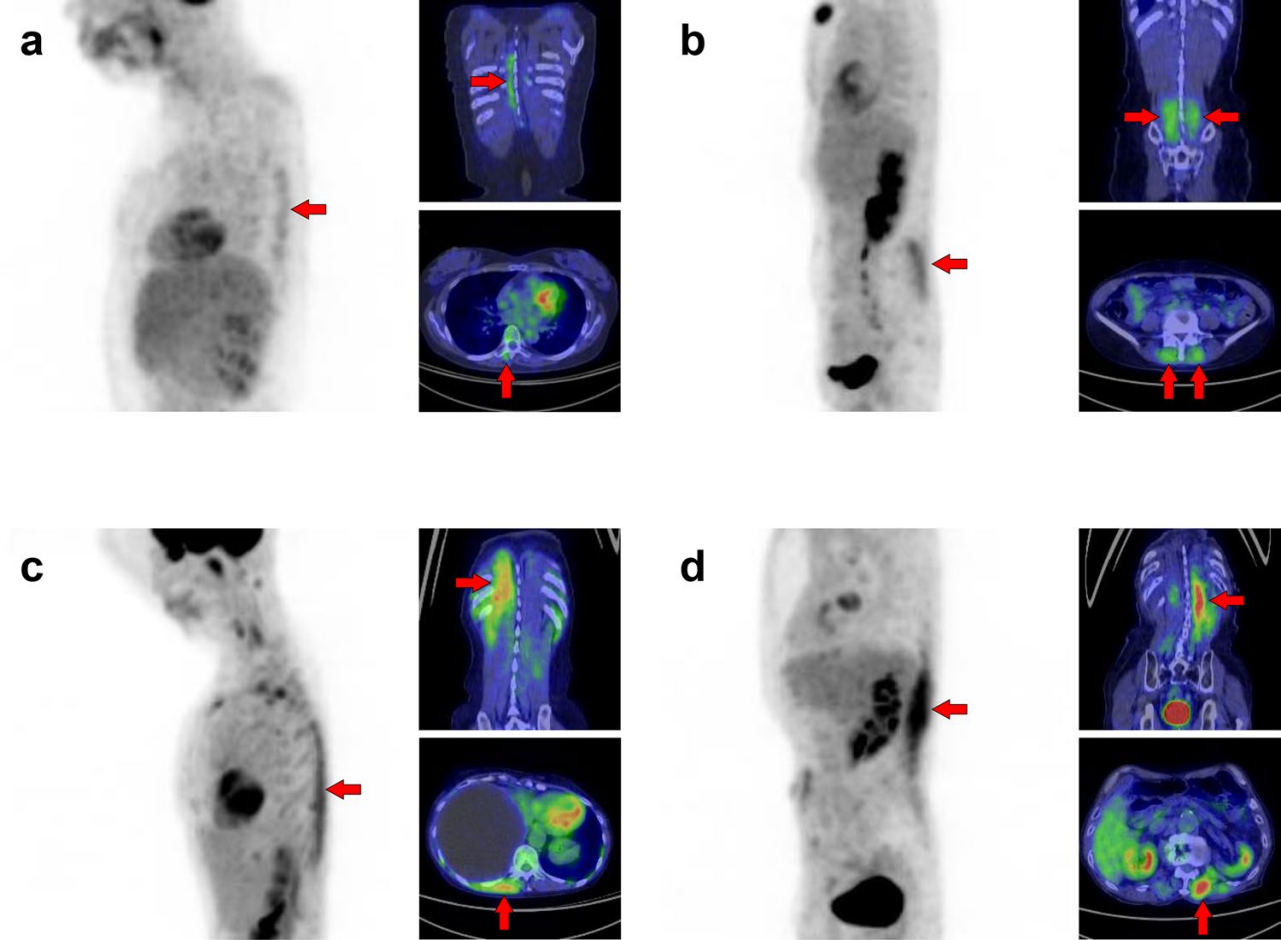
*Transversospinales muscles and erector spinae muscles.* In the thoracoabdominal area, intense uptake is frequently observed in the back muscles. We present two cases of the uptake in the transversospinales (semispinalis and multifidus) (**a** and **b**, arrows) and two cases of that in the erector spinae (longissimus and iliocostalis) (**c** and **d**, arrows)

Excessive walking increases [^18^F]FDG uptake in the lower extremities [14]. However, we occasionally observed asymmetrical or single muscle uptake, which makes it difficult to interpret the mechanism. Older individuals generally have a higher muscle glucose metabolism than younger individuals with the same movements [15]. Therefore, some specific muscles may be overloaded as skeletal motor function declines, which is likely to be a factor in the increased FDG uptake in a solitary muscle. Further research is required to elucidate the underlying mechanism. Figure 20 illustrates a schema of muscles in the abdomen, pelvis, and femur regions where physiological uptake is frequently observed. Among them, we illustrated the psoas major muscle (Fig. 21), iliacus muscle (Fig. 22), gluteus medius and gluteus maximus muscles (Fig. 23), quadratus femoris, pectineus, and obturator externus muscles (Fig. 24), semitendinosus and gracilis muscles (Fig. 25), adductor longus and adductor brevis muscles (Fig. 26), quadriceps femoris muscles, tensor fasciae latae muscle, and sartorius muscle (Fig. 27), and transversospinales muscles and erector spinae muscles (Fig. 28).

## Conclusion

Various specific patterns of solitary or asymmetrical skeletal muscle uptake can be observed on routine [^18^F]FDG-PET scans. Therefore, we should avoid misunderstanding these uptakes as malignant tumors or inflammatory diseases by understanding the typical physiological patterns.
